# Study of the genetic variability in a Parkinson's Disease gene: EIF4G1

**DOI:** 10.1016/j.neulet.2012.04.033

**Published:** 2012-06-14

**Authors:** Arianna Tucci, Gavin Charlesworth, Una-Marie Sheerin, Vincent Plagnol, Nicholas W. Wood, John Hardy

**Affiliations:** a Department of Molecular Neuroscience and Reta Lila Weston Laboratories, Institute of Neurology, Queen Square House, Queen Square, London WC1N 3BG, United Kingdom; b UCL Genetics Institute, University College London, Darwin Building, Gower Street, London WC1E 6BT, United Kingdom

**Keywords:** Parkinson's Disease, EIF4G1

## Abstract

Chartier-Harlin and colleagues [2] recently reported mutations in the eukaryotic translation initiation factor 4-gamma (EIF4G1) gene in families with parkinsonism. Large-scale screening found two mutations (p.R1205H and p.A502V) only in affected individuals, although their relative frequency was very low. The aim of this study was to investigate EIF4G1 parkinsonism-related variants in two separate cohorts and study coding variability across the gene. We first screened a series of familial Parkinson's Disease (PD) patients in an attempt to confirm previous results by showing segregation. Then, to determine the extent of coding variation in the gene, we first screened a cohort of sub-Saharan African individuals from the Centre d’Etude du Polymorphisme Humain – Human Genome Diversity Cell Line Panel (HGDP) [1] and then analyzed data from 5350 individuals National Heart, Lung, and Blood Institute (NHLBI) exome sequencing project. We failed to identify any PD-related mutations in the familial samples. Conversely we found the p.A502V variant in the NHLBI population. We observed a high number of coding polymorphism in the exons where the two PD variants have been previously reported. We conclude that either EIF4G1 variants are an extremely rare cause of familial PD in Caucasian cohorts, or that A502V is in fact a rare benign variant not involved in PD aetiology. Our data also suggests that the protein can tolerate some extent of variability particularly at this point of the gene.

## Highlights

► Mutations in the EIF4G1 gene have been found in patients with Parkinson's Disease (PD) [2]. ► We investigate the mutations in a familial PD cohort and in sub-Saharian Africans and Europeans. ► We failed to identify any mutation in the PD cohort. We identify the p.A502V (previously associated to PD) in Caucasians. ► Mutations in the EIF4G1 gene are a very rare cause of PD in European individuals. ► The protein can tolerate some extent of genetic variability.

Recently, a new gene (EIF4G1) has been identified in one family with autosomal dominant late-onset Parkinson's Disease [2]. Linkage was ascribed to a region at chromosome 3q26–28 containing approximately 159 genes. Sequence analysis found only one novel coding variant (p.R1205H in the EIF4G1 gene) which segregated with disease, which was absent in 4050 controls and which was evolutionary conserved in mammals. Screening a cohort of about 4800 PD cases (familial and sporadic) identified nine additional patients of the p.R1205H mutation. Further molecular analysis of the EIF4G1 gene in a large case–control cohort (4500 cases and 3800 controls) identified another novel missense mutation (p.A502V) in three PD individuals, which was not found in controls. These data indicate that these variants are extremely rare in the PD population (0.2% for p.R1205H and 0.06% for p.A502V). Assignment of pathogenicity can be difficult when variants are very rare. With this background, we screened 150 familial PD cases from our UK familial Parkinson's Disease series, in which we have previously identified LRRK2, VPS35 and SNCA mutations [5,7] in order to determine whether we could provide further that this gene is indeed a PD-related locus. We also assessed these coding positions in a set of African samples (Table 1) from the Human Diversity series – a standard panel of African samples [1], as African samples have the greatest diversity and offer a rapid route to the identification of benign polymorphisms [4]. Briefly, exon 8 and exon 22 of the EIF4G1 gene (NM_182917.3) were PCR amplified and sequenced in the two cohorts for a total of 114 African samples and 150 familial PD cases.

To obtain a more exhaustive description of the pattern of variability in that gene we also extracted genotype data from the NHLBI exome sequencing project (Exome Variant Server, NHLBI Exome Sequencing Project (ESP), Seattle, WA (http://evs.gs.washington.edu/EVS/) [January 2011], which includes exome data for 3500 American individuals of European descent and 1850 African American. Frequencies were computed using VCFtools. We used the software ANNOVAR [8] to annotate the function of the variants.

We failed to identify any mutation previously reported to be associated with PD our familial cohort, but we identified one coding change (P486S) in two PD individuals. The P486S variant is reported in dbSNP (rs112545306). Interestingly it has been observed in African-Americans (http://snp.gs.washington.edu/EVS), with a frequency of 0.15%.

We identified six non-synonymous changes in exon 8 and in exon 22 in the African individuals. Of these, one is a novel change (P382L), the others are variants recently reported in dbSNP and found mainly in African populations (http://snp.gs.washington.edu/EVS). To predict the impact on protein function of these non-synonymous variants, we performed an in silico analysis using the software PolyPhen and SIFT [6] and all were predicted to be benign (Table 2).

Analysis of the NHLBI samples allowed us to detect the A502V variant in two European-American individuals (frequency of 0.02%). We identified 95 nonsynonymous SNP over 32 exons in total (NM_182917.3). Of note, 36 of them are located in exon 8 and exon 22 (Table 3). To investigate coding variability across the EIF4G1 gene we extracted the data from the NHLBI dataset and computed the average number of pairwise amino acid differences between two randomly selected European-American haplotypes from the NHLBI dataset (Methods). On average two such EIF4G1 sequences diverge by 0.45 aminoacids. 82% of this variability (0.37 amino-acid differences) locates to exon 8, where the A502V lies. 9.8% of this variability locates to exon 22 (0.098 amino-acid differences). Combined with the identification of six coding variants in exons 8 and 22 in African samples, these data are consistent with a more limited selective pressure and a higher sequence variability in this region of the EIF4G1 protein.

These data, combined with the presence of the A502V in the NHLBI population with 0.02% frequency and our failure to identify any PD mutation carrier in our familial cohort, are consistent with the interpretation that either EIF4G1 variants are an extremely rare cause of familial PD in Caucasian cohorts, or that A502V is in fact a rare benign variant not involved in PD aetiology.

## Role of the funding source

This study was supported by Parkinson's Disease Society (AT) and by the Medical Research Council and Wellcome Trust Disease Centre (grant WT089698/Z/09/Z). DNA extraction work was undertaken at University College London Hospitals, University College London, who received a proportion of funding from the Department of Health's National Institute for Health Research Biomedical Research Centres funding.

## Disclosure statement

Arianna Tucci, Gavin Charlesworth, Una-Marie Sheerin, Vincent Plagnol, Nick Wood, John Hardy report no disclosures.

## Figures and Tables

**Table 1:** Number of African samples studied per population and geographic regions.

No. of samples	Population	Geographic origin
35	Biaka Pygmy	Central African Republic
15	Mbuti Pygmy	Democratic Republic of Congo
12	Bantu N.E.	Kenya
7	San	Namibia
26	Yoruba	Nigeria
24	Mandenka	Senegal
8	Bantu S.E. Pedi	South Africa

**Table 2:** EIF4G1 coding variant detected in the African cohort (NM_182917.4).

Nucleotide change	Protein change	SNP accession number	Frequency	Population	Effect (SIFT)
c.870G>A	M290I	rs144947145	0.01	San, Bantu SE	Tolerated
c.913C>T	R305C	rs116508885	0.01	Youruba	Tolerated
c.932A>G	Y311C	rs16858632	0.03	Bantu SW, Bantu NE, Yoruba, Mandenka	Tolerated
c.1145C>T	P382L	NA	0.004	San	Tolerated
c.1429G>A	E477K	rs145228718	0.004	Mandenka	Tolerated
c.3918G>A	R1216H	rs34086109	0.004	Biaka Pygmy	Tolerated

**Table 3:** EIF4G1 coding variants present in NHLBI Exome Sequencing Project (NM_182917.4). EA = European American population. AA = African American population.

Nucleotide change	Protein change	SNP accession number	Frequency (EA)	Frequency (AA)	Exon
c.C71T	P24L		0.000142	0	2
c.C167G	A56G		0.000142	0	3
c.C211T	P71S	rs113810947	0.000285	0	3
c.282C>G	I94M		0	0.000268	3
c.451C>G	V151L		0.000143	0	5
c.481A>G	A161T	rs13319149	0.996722	0.999465	5
C.602G>A	R201H	rs34838305	0.000427	0	6
C.608C>T	A203V		0.000142	0	6
C.704G>A	R235Q	rs144543953	0.000142	0.000268	8
C.731G>A	R244Q	rs147855566	0.000142	0	8
C.779C>T	S260L		0.000142	0	8
c.821C>T	P274L	rs139626338	0	0.000268	8
c.870G>A	M290I	rs144947145	0	0.001338	8
c.913C>T	R305C	rs116508885	0	0.00321	8
c.914G>A	R205H	rs151151194	0.000142	0.000268	8
c.926A>G	E309G		0.000142	0	8
c.932A>G	Y311C	rs16858632	0.000427	0.059658	8
c.1001A>C	P334Q		0.000285	0	8
c.1013C>T	S338F	rs139021806	0	0.000268	8
c.1036C>A	Q346K		0	0.000268	8
c.1054G>T	A352S		0.000142	0	8
c.1063A>G	T355A		0	0.000268	8
c.1064C>T	T355I		0	0.000268	8
c.1142C>G	A381G	rs142095694	0.000142	0	8
c.1256G>T	S419I	rs138207269	0	0.000535	8
c.1294A>G	M432V	rs2178403	0.759544	0.943553	8
c.1298C>T	A433V	rs145998921	0.000142	0	8
c.1309A>G	I437V	rs144222028	0	0.000268	8
c.1316C>A	S439Y	rs148709174	0.000142	0	8
c.1331C>T	T444M	rs143014570	0	0.000268	8
c.1352C>A	P451Q	rs147419996	0.000142	0	8
c.1429G>A	E477K	rs145228718	0	0.000268	8
c.1456C>T	P486S	rs112545306	0.00057	0.000803	8
c.1505C>T	A502V	rs111290936	0.000285	0	8
c.1610C>T	A537V		0	0.000268	10
c.1648G>C	A550P	rs111924994	0.001994	0.000535	10
c.1679G>A	G560D	rs149685875	0.000142	0	10
c.1696C>T	R566C	rs145521479	0.000142	0.000268	10
c.1700C>A	P567H	rs140212150	0	0.000268	10
c.1754A>C	E585A		0	0.000268	10
c.1801T>C	W601R	rs145247318	0	0.000268	11
c.1831C>T	R611C		0	0.000268	11
c.1980A>G	I660M		0.000142	0	12
c.1982A>G	N661S	rs145780534	0.000142	0	12
c.2096G>C	G699A		0.000142	0	13
c.2114C>G	S705C	rs141054452	0.000142	0	13
c.2152G>C	A718P	rs111396765	0.001567	0.000268	13
c.2225C>T	T742M	rs147678593	0	0.004013	13
c.2276A>G	Q759R		0.000142	0	14
c.2278G>C	D760H	rs142947014	0.000142	0	14
c.2386A>G	K796E		0	0.000268	14
c.2419A>G	I807V	rs62287499	0.000427	0	14
c.2488A>T	T830S	rs111500185	0.000285	0	15
c.2612A>C	E871A		0.000142	0	15
c.2671A>G	I891V		0	0.000268	16
c.2882A>G	N961S	rs191888688	0	0.000268	17
c.3187C>T	R1063C		0.000142	0	19
c.3221C>T	T1074I	rs146433145	0.000142	0	19
c.3343C>T	R1115C	rs150054202	0	0.000268	21
c.3428A>G	Q1143R	rs145414660	0	0.000268	21
c.3482G>A	R1161H	rs139135683	0.000427	0	22
c.3511C>T	R1171C	rs141684202	0	0.000268	22
c.3529C>T	R1177C		0	0.000268	22
c.3580C>T	R1194W		0.000142	0	22
c.3584G>A	S1195N		0.000142	0	22
c.3592C>T	R1198W	rs113388242	0	0.000268	22
c.3617G>A	R1206H	rs112176450	0.000285	0	22
c.3649C>T	R1207C		0.000143	0	22
c.3650G>A	R1217H	rs34086109	0	0.010433	22
c.3652G>A	G1217R	rs138270117	0	0.000268	22
c.3686C>T	P1299L		0	0.000268	23
c.3688C>G	P1230A	rs35629949	0.005842	0.001606	23
c.3701T>C	L1234P	rs2230570	0.021937	0.080257	23
c.3743A>G	K1248R		0.000142	0	23
c.3773A>G	N1258S	rs73053766	0	0.001873	23
c.3935C>T	S1312F		0	0.000268	24
c.3937A>G	T1313A	rs144570332	0.000142	0.000803	24
c.3988A>G	M1330V	rs112809828	0.000285	0	25
c.4067T>C	M1356T	rs144059151	0.000855	0	25
c.4068G>C	M1356I	rs145975905	0.000285	0	25
c.4081A>G	R1361G	rs139793721	0	0.000268	25
c.4106C>T	P1369L	rs142064428	0	0.000803	26
c.4184C>T	T1395M	rs112441721	0	0.000268	27
c.4201G>A	G1401R	rs149821418	0.000142	0.000803	27
c.4229A>C	E1410A	rs141776790	0	0.000268	27
c.4259A>G	E1420G		0	0.000268	27
c.4292C>T	S1431L		0.000142	0.000268	28
c.4300C>T	P1434S	rs147696097	0.000142	0	28
c.4379G>A	R1460Q		0.000142	0	28
c.4399G>A	A1467T	rs148270724	0.000142	0	29
c.4433C>T	T1478M	rs141379472	0.000142	0	29
c.4454C>T	T1485M		0.000142	0	29
c.4486A>T	T1496S		0	0.000268	29
c.4712C>T	A1571V	rs144462594	0.000142	0	31
c.4772G>A	R1591H		0.000142	0	31
